# Generation of diffraction-free optical beams using wrinkled membranes

**DOI:** 10.1038/srep02775

**Published:** 2013-09-27

**Authors:** Ran Li, Hui Yi, Xiao Hu, Leng Chen, Guangsha Shi, Weimin Wang, Tian Yang

**Affiliations:** 1 University of Michigan - Shanghai Jiao Tong University Joint Institute, National Key Laboratory of Nano/Micro Fabrication Technology, Key Laboratory for Thin Film and Microfabrication of the Ministry of Education, Shanghai Jiao Tong University, Shanghai 200240, China; 2 These authors contributed equally to this work.

## Abstract

Wrinkling has become a well developed bottom-up technique to make artificial surface textures in about the last decade. However, application of the optical properties of long range ordered wrinkles has been limited to one dimensional gratings to date. We report the demonstration of macroscopic optical focusing using wrinkled membranes, in which concentric wrinkle rings on a gold-PDMS bilayer membrane convert collimated illuminations to diffraction-free focused beams. Beam diameters of 300–400 μm have been observed in the visible range, which are dominantly limited by the eccentricity of the current devices. Based upon agreement between theoretical and experimental results on eccentricity effects, we predict a decrease of the beam diameter to no more than around 50 μm, if eccentricity is eliminated.

Wrinkling and buckling are commonplace phenomena in nature, e.g. skin wrinkles, finger prints, surface patterns of plants and folded mountains^1^,^2^. Understanding and modeling of wrinkle formation have greatly advanced in about the last decade. The most commonly employed model is a bilayer membrane which is composed of a rigid and thin elastic surface layer on top of a soft and thick elastic substrate^3^,^4^,^5^,^6^,^7^. When the surface layer is under compressive strain by contraction of the substrate, the membrane spontaneously wrinkles to minimize its total energy which contains the bending energy and the stretching energy. Both theoretical modeling and experiments have shown that the wrinkle period is a function of the thicknesses, the Young's moduli and the Poisson's ratios of the surface and substrate layers, and that the wrinkle amplitude scales with the wrinkle period and the imposed strain. Several basic periodic wrinkle patterns have been theoretically predicted and experimentally produced, which include line, square, triangular, herringbone and cap-like patterns^3^,^4^,^6^,^7^,^8^,^9^,^10^. To date, a variety of methods to fabricate artificial wrinkles at the micrometer and nanometer scales have been reported, with good control of the periods and amplitudes, and with more complicated patterns^3^,^4^,^9^,^11^,^12^,^13^,^14^,^15^,^16^,^17^,^18^.

The controllable and tunable formation of wrinkle patterns has attracted a lot of interest, since it is a very cost effective bottom-up technique to make artificial surface textures and to engineer the surface and bulk properties of materials. Many applications of wrinkles have been reported^2^, including measurement of thin film properties^19^,^20^, control of cell proliferation^21^, microcontact printing masters^22^, modification of surface wettability and adhesion^15^,^23^,^24^, microfluidic devices and nanoparticle assembly^11^,^25^, flexible electronics and optics^26^,^27^,^28^,^29^, and tunable materials^30^,^31^,^32^,^33^. In the area of optics, one dimensional periodic wrinkles have been used as optical gratings as early as the invention of artificial formation of ordered wrinkles^4^,^19^,^34^. However, although it has been anticipated that the periodic nature of wrinkles could be interesting for the facile production of a range of optical devices^2^, except for one dimensional gratings, applications of the optical properties of wrinkles have not been explored until recently. These applications include microlens fabrication^35^, enhancement of the light extraction efficiency from organic light emitting diodes^36^, and increasing the light harvesting efficiency of polymer photovoltaic devices^37^. But these applications are still not based upon the optical properties of regular and long-range ordered wrinkle patterns.

In this paper, we report the demonstration of macroscopic optical focusing membranes based upon long-range ordered two dimensional wrinkles. These thin membranes have the same functions as axicon lenses, which have conical surfaces that are rotationally symmetric. They both convert collimated optical beams to diffraction-free focused beams in the focus region, which are Bessel beams in the ideal situation, and to rings in the farther space or the divergence region. Axicons have been widely used in corneal surgery, optical trapping, optical coherence tomography, telescopes, laser manufacturing, etc.^38^,^39^,^40^,^41^. While this paper reports axicon-like performance, we expect that a family of wrinkled membranes with different optical focusing functions could be achieved by strain distribution engineering. They will have advantages over the bulky glass optics counterparts in terms of low fabrication cost, light weight and applicability to curved surfaces. In addition, large area wrinkled membranes could be easily and cost effectively fabricated, while glass optics and lithographically fabricated zone plates are usually limited in size. However, as a bottom-up process, wrinkling may not achieve the uniformity and precision of top-down fabrication as used in glass optics manufacturing and lithography. In this paper, we identify the limiting factor for the current devices' focusing performance, and predict a focal spot size of no more than around 50 μm in the visible range after eliminating eccentricity.

## Results

### Devices and the fabrication method

**Figure 1:**
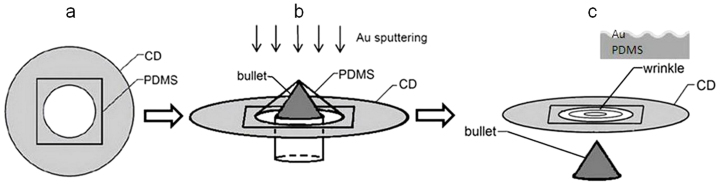
Fabrication procedure for a Au-PDMS bilayer membrane with concentric wrinkle rings. (a) A piece of flat PDMS film is glued onto a CD. (b) The PDMS film is pulled at its center by a bullet shaped metal piece, followed by the sputtering of a thin film of Au. (c) The bullet is withdrawn, and the Au-PDMS bilayer membrane becomes a flat membrane with concentric wrinkle rings. The inset shows a cross section of the wrinkles.

**Figure 2:**
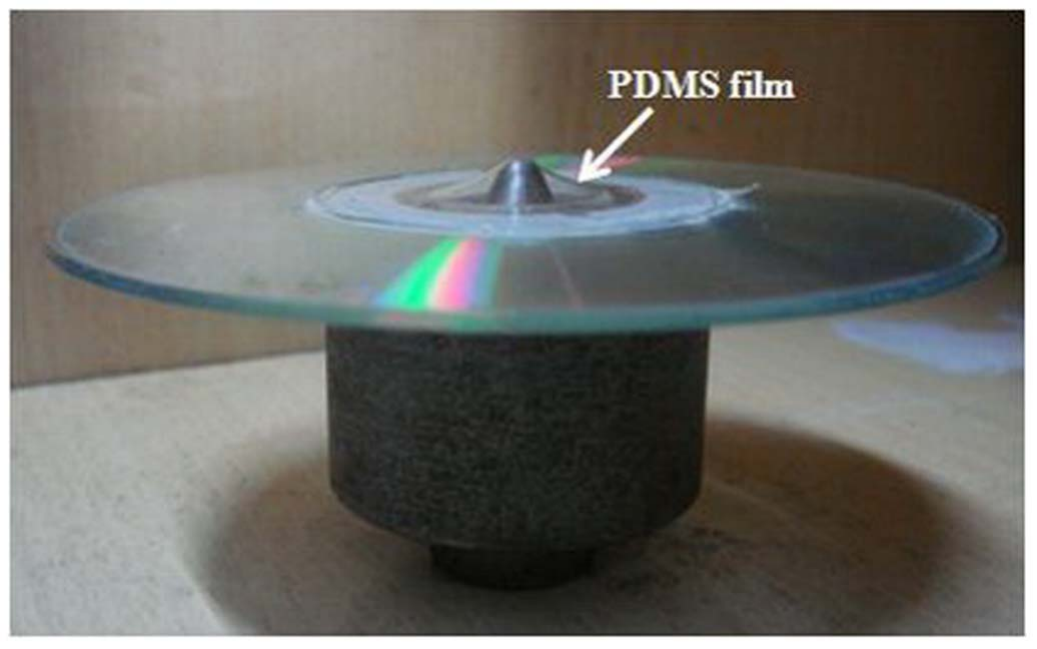
A piece of PDMS film on a CD and being pulled at its center.

Each of our optical focusing membranes consists of a set of concentric wrinkle rings in a gold(Au)-polydimethylsiloxane(PDMS) bilayer thin membrane. In the ideal situation, the wrinkle rings would have a uniform period. The whole fabrication procedure and a schematic illustration of the final device are shown in Figure 1. First, we placed a piece of PDMS film flatly on top of a hard substrate which had a circular hole in the center, which in our experiment was a Compact Disc (CD). The PDMS film we used was 170 μm thick. Uncured PDMS mixture, in the form of viscous fluid, was applied between the CD and the PDMS film as an adhesion agent. A three hour oven curing at 60°C made the PDMS film firmly bound to the surface of the CD. In order to form concentric and uniformly periodic wrinkle rings, radially oriented and uniformly distributed strain should be applied, the directions of strain being perpendicular to the expected wrinkles. This was achieved by pulling the center of the freely hanging PDMS film as shown in Figure 2. In the pulling device, a bullet-shaped metal piece with a semi-spheroidal tip pushed the center of the PDMS film from below to stretch the film by 40%. We moved the bullet by rotating a screw underneath it, so that the bullet itself was not rotating in order to avoid friction with the PDMS film and to reduce unwanted strain in the azimuthal direction. To further eliminate the friction between the bullet and the PDMS layer, methanol was applied between them as a lubrication fluid. Then we coated a thin layer of Au onto the PDMS film, in a sputter coater which is commonly used for Scanning Electron Microscopy (SEM). Finally, we withdrew the bullet, so that the radial stress between the Au layer and the PDMS substrate induces wrinkles along the azimuthal direction to reach the lowest mechanical energy state, whose period and amplitude values can be controlled via the thickness of the Au layer and the prestretch of the PDMS film respectively.

**Figure 3:**
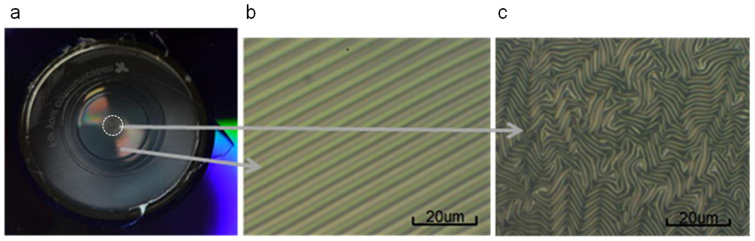
A piece of Au-PDMS bilayer membrane with concentric wrinkle rings. (a) The whole membrane on a CD, observed under room light. (b) Part of the concentric wrinkle rings under an optical microscope. (c) Part of the opaque central part of the membrane under an optical microscope, which corresponds to the region within the white dashed circle in Figure 3a.

Figure 3a shows the photo of a fabricated device under room light. The shiny colors on the membrane came from the diffraction of room light by the periodic wrinkles, while the central part of the membrane (within the white dashed circle) that had been in contact with the bullet looks opaque. As shown by the optical micrograph in Figure 3b, this device contains concentric wrinkle rings centered around the bullet pushing point, with a locally uniform period. The wrinkles form a regular and long-range ordered pattern over the 14 mm diameter CD hole, except for the part which had been in contact with the bullet as shown in Figure 3c. The nonuniformly oriented and smaller period patterns in the central opaque region result from friction between the membrane and the semi-spheroidal tip of the bullet, which impedes the free movement and wrinkling of the membrane. The wrinkle pattern gradually evolves from Figure 3c to Figure 3b as the observation spot moves from the center out of the opaque region, since the center has been in contact with the bullet tip for a longer time than the outer parts of the opaque region during the bullet withdrawal procedure.

### Optical focusing performance

**Figure 4:**
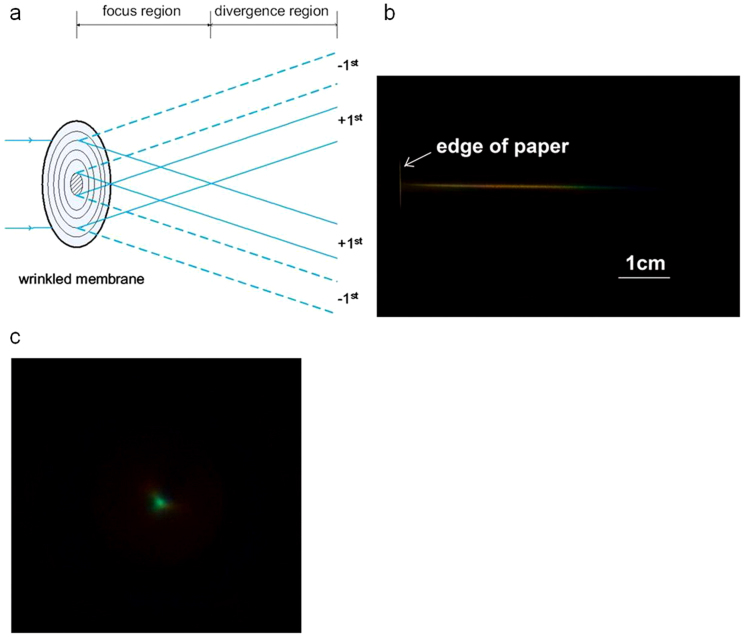
Optical characterization of the focusing performance of a wrinkled membrane. (a) A schematic illustration of the optical experiment. The wrinkled membrane is illuminated with a collimated beam of light at normal incidence onto the Au side. The central part of the membrane is opaque. The +1^st^ order diffraction bends light to the center, and the −1^st^ order diffraction bends light away from the center. The focus region and divergence region of the device are schematically indicated. (b) A side view of the diffraction-free beam in the focus region, on a piece of white paper. The front edge of the paper is about 1 mm away from the membrane. (c) A front view of the diffraction-free beam in the focus region, at a distance of 3.5 cm from the membrane, on a piece of white paper. The focal spot is 300–400 μm in diameter.

We have characterized the focusing performance of the wrinkled membranes by illuminating them with a collimated beam of light at normal incidence. There had not been observable changes in their focusing performance half a year after the devices were fabricated. The illuminating beam was from a broadband halogen lamp and 11 mm in diameter. A schematic illustration of the experiment is plotted in Figure 4a. It shows that the +1^st^ order diffraction off the concentric and uniformly periodic wrinkle rings is analogous to the refraction from the conical surface of an axicon, whose interference with itself produces a diffraction-free focused beam in the focus region of the device. In the divergence region, the +1^st^ and −1^st^ orders of diffraction form two optical rings respectively, separated by the opaque central part of the membrane. In Figure 4b and 4c, we took side and front views of the focused optical beam with a piece of white paper, for the device shown in Figure 3. A five centimeter long, 300–400 μm in diameter, and diffraction-free rainbow beam was observed. The beam diameter is predominantly limited by the eccentricity of the sample, which will be explained later.

The optical characterization results have been compared with theoretical modeling. The wrinkled membrane in Figure 3 has an average period around 4.7 μm and a Au layer thickness around 13 nm, the latter measured by Atomic Force Microscopy. By diffraction theory, a concentric ring grating (with a good central part) that has a 4.7 μm period will focus a 11 mm diameter and 0.5 μm wavelength (blue) incident beam to a 5.2 cm long line, and a 11 mm diameter and 0.7 μm wavelength (red) incident beam to a 3.7 cm long line. This is in good agreement with the experimentally observed rainbow line shown in Figure 4b. The amplitude of wrinkles determines the wavelength where diffraction is most efficient. The peak-to-peak amplitude of the wrinkles in this device is around 0.7 μm, according to Atomic Force Microscopy (AFM) as shown in Supplementary Figure S1. Finite difference time domain (FDTD) calculation of a one dimensional sinusoidal grating's diffraction efficiency is plotted in Supplementary Figure S2, which takes the same material, period, and amplitude as the wrinkle-ring device. The calculation shows a maximum +1^st^ order diffraction efficiency of 24% for blue light. The effect of optical loss introduced by the Au film is also presented in the figure, which shows that by replacing gold with transparent surface layer materials, e.g. oxidized PDMS, the diffraction efficiency will be further increased to 34%^4^. The focal depth and peak efficiency wavelength can be tuned straightforwardly by tuning the period and amplitude of the wrinkles. The period scales linearly with the thickness of Au film, while the amplitude-to-period ratio scales with the square root of compressive strain^5^.

### Period eccentricity and limit of focusing ability

Further decreasing the diffraction-free beam' diameter is crucial for many applications. Just as many other bottom-up processes, wrinkle structures contain defects and non-uniformity, which are analogous to lens aberrations and result in focal spots larger than the diffraction limit. In this section, we report a study on this problem. We find that the microscopic local defects and non-uniformity only impose a minor broadening of the focal spot size that is not more than around 50 μm, while the macroscopic period eccentricity is the dominant reason for the large beam diameters in our experiments.

**Figure 5:**
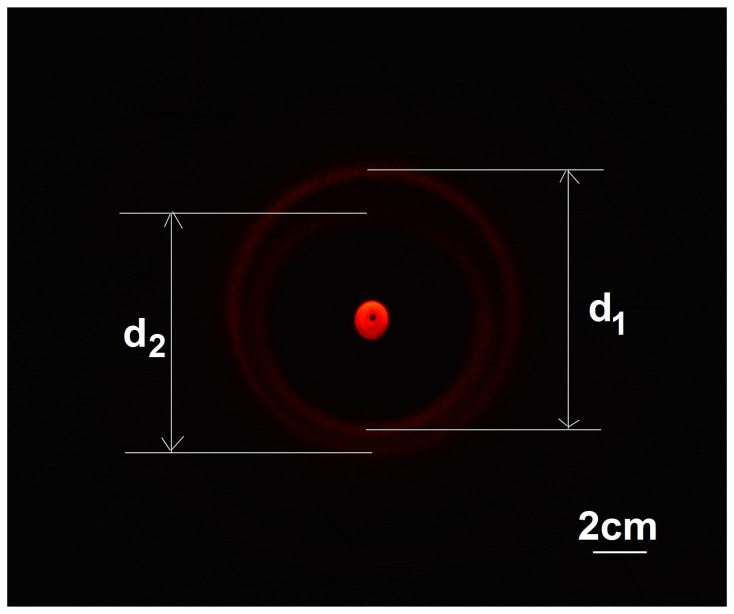
Optical rings in the divergence region of a wrinkled membrane. By illuminating a wrinkled membrane with a collimated beam from a He-Ne laser at normal incidence, a pair of rings in the divergence region were observed. The bright spot in the center was non-diffracted laser light. The picture was taken on a piece of white paper at a distance of 33 cm from the membrane.

It has been observed for all of our wrinkle-ring devices that the period is different at different azimuthal angles, which we call “period eccentricity” in this paper. Period eccentricity has been confirmed by both microscopy and by measuring the optical diffraction rings in the divergence region. The diffraction ring pattern renders a clear quantitative description of period eccentricity and is discussed as follows. In this experiment, we illuminated the same device as shown in Figure 3 with a collimated beam from a He-Ne laser at normal incidence, which was around 12 mm in beam diameter and 633 nm in wavelength. Figure 5 shows a front view of the diffraction ring pattern at a distance of 33 cm from the membrane, which contains a pair of rings. The inner ring comes from the +1st order diffraction, the outer ring comes from the −1st order diffraction, and the dark area between the rings comes from the opaque central part of the membrane, as illustrated in Figure 4a. By substituting the double ring pattern into equation (1) in the Methods section, we calculated the period value at each azimuthal angle, which is shown in Supplementary Figure S4. For example, by the lengths d_1_ and d_2_ in the double ring pattern in Figure 5, we calculated the period between the pushing point and the top CD hole edge to be 4.5 um, and the period between the pushing point and the bottom CD hole edge to be 5.0 µm.

**Figure 6:**
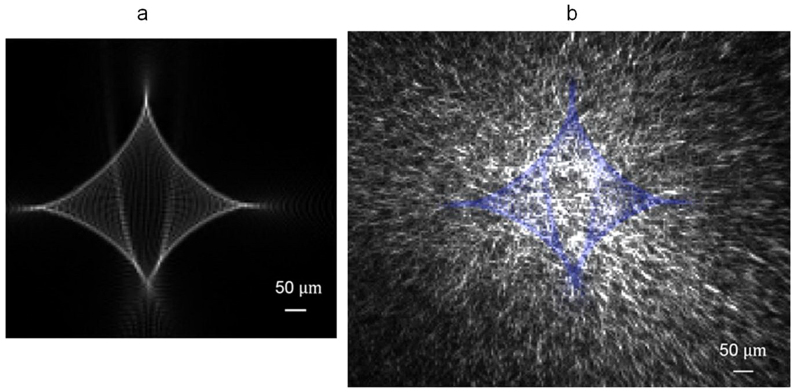
Calculated and measured He-Ne laser focal spot profiles off a wrinkled membrane. (a) Calculated electric field intensity profile at a distance of 3 cm from the membrane, when azimuthal variation of period is the only imperfectness. (b) An image of the focal spot taken by a monochrome CMOS camera chip, with the calculated features from (a) overlapping on top in blue. The distance between the CMOS chip and the membrane is 3–3.5 cm.

Then we calculated the electric field intensity profile of a focused beam off an imaginary wrinkle-ring device, whose only imperfectness is the period eccentricity as in Supplementary Figure S4, and which has a sinusoidal wrinkle shape and a uniform wrinkle amplitude of 0.7 μm. The calculation is based upon the Huygens-Fresnel principle, more details included in the Methods section. Figure 6a shows that, when this imaginary device is illuminated by a collimated He-Ne laser at normal incidence, the focal spot at a distance of 3 cm is 300–400 μm in size, with rhombus-shaped sides and other line-shaped features.

For comparison, an experimental image of the focal spot taken by a monochrome CMOS camera chip is shown in Figure 6b, with the calculated features for the imaginary device overlapping on top. Each pixel of the CMOS chip is a 5.2 μm × 5.2 μm square. The calculated features and the experimental image match each other fairly well, both containing four bright arc segments on the circumference and two bright feature lines in the center, with almost the same orientations and dimensions as each other, except that the top right arc segment in the experimental image was not clearly observed. This comparison clearly indicates that period eccentricity dominantly broadens and shapes the focused beam to what have been observed experimentally. The line-shaped features in the experimental image are around 50 μm wide, which is a combined consequence of all the other imperfectnesses expect period eccentricity, including microscopic local defects, non-uniformity and other macroscopic errors.

## Discussion

The significant period eccentricity in our devices is most probably due to a non-uniform Au layer thickness, the reason of which is still under exploration. If we can eliminate this effect by improving our fabrication, we expect to obtain focal spots as small as around 50 μm, which will find many applications such as optical trapping, optical coherence tomography, telescopes, laser manufacturing, etc. In addition, we expect other types of wrinkled membrane focusing devices, e.g. Fresnel lenses that focus to a single spot, to be fabricated by strain distribution engineering. This can be done, for example, by placing a circular shutter centered above the membrane during Au deposition, so that the thickness of Au film varies across the membrane as designed, and specific period-versus-radial-distance wrinkle patterns can be achieved. These wrinkled membrane focusing devices have advantages over bulky glass optics and lithographically patterned zone plates in terms of low cost, light weight, flexibility and suitability for large area fabrication.

In conclusion, we have reported, as far as we know, the first demonstration of macroscopic optical focusing using long-range ordered wrinkled membranes. A concentric wrinkle ring pattern was formed in a 13 nm Au – 170 μm PDMS bilayer membrane, with a period around 4.7 μm. The device focuses collimated illuminating beams, which have diameters of around 1 cm, to diffraction-free beams which have diameters of 300–400 μm and focal lengths of 4–5 cm in the visible range. Further, by studying the diffraction rings and comparing the calculated and experimentally measured focal spot profiles, we have identified period eccentricity as the dominant focal spot broadening and shaping factor for our current devices. If device eccentricity can be eliminated, we predict the focal spots to be as small as around 50 μm. We anticipate the wrinkled membranes to become a viable solution where low cost, light weight and flexible focusing lenses are in need.

## Methods

### Preparation of PDMS films

We prepared the PDMS (Dow Corning Sylgard-184) using an oligomer to curing agent ratio of 10:1 by weight. The PDMS mixture was degassed in a low vacuum environment for 20 minutes, then spin-coated onto a copper coated silicon wafer, and cured at 60°C in an oven for three hours, so that a flat and solid PDMS film was formed. The copper coating on the silicon wafer facilitates lifting the PDMS film from the wafer.

### Calculation of beam profiles under eccentricity

We calculated the electric field intensity profiles of the focused beam when period eccentricity is the only imperfectness of the device, i.e. when the wrinkle period is a constant in each radial direction, as follows.

In our model, the wrinkled membrane is illuminated with a collimated He-Ne laser at normal incidence. The parameters used in the calculation are defined in Supplementary Figure S3. We manually defined the center lines of the optical rings in Figure 5, as shown in Supplementary Figure S3b. Accordingly we calculated the period, *L*, for each azimuthal angle, *θ*, by the following equation, with the calculation result shown in Supplementary Figure S4. 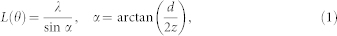
where *λ* = 633 nm and *z* = 33 cm.

The electric field amplitude transmittance function of the wrinkled membrane, *U_t_*, was taken to be the following. 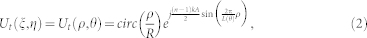
where *ρ* is the radial coordinate, *R* is the radius of the membrane, *n* = 1.4 is the refractive index of PDMS, *A* = 0.7 μm is the peak-to-peak amplitude of the wrinkles, and 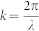 is the wave vector. Here we have assumed that the wrinkles have a sinusoidal shape, that *U_t_* has a uniform amplitude of 1, that *U_t_*′s phase changes from 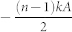 to 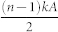 in each wrinkle period, and that *U_t_*′s phase is 0 at the origin along all radial directions (no azimuthal phase variation).

Then the scalar electric field on the observation point, *U*(*x*,*y*,*z*), was calculated by the Huygens–Fresnel principle as follows. 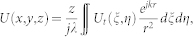

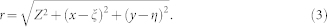


Finally, the electric field intensity profile on the observation plane *z* *=* *z_0_* was plotted as |*U*(*x*,*y*,*z_0_*)|^2^, as shown in Figure 6a, where *z_0_* = 3 cm. One point per 2 μm × 2 μm area on the observation plane has been calculated, and the nearest 3 × 3 points have been averaged.

## Author Contributions

R.L., L.C., G.S. and W.W. fabricated the devices. H.Y. and T.Y. performed the optical measurements. X.H. finished the calculations. T.Y. led the reported work and prepared the manuscript.

## Supplementary Material

Supplementary Information Supplementary Information
