# Supplementary material for: Generation of diffraction-free optical beams using wrinkled membranes
Source: Sci Rep. 2013 Sep 27;3:2775. doi: 10.1038/srep02775 (PMC3784948; doi:10.1038/srep02775)
Supplement: Supplementary Information [file srep02775-s1.pdf]

# Supplementary Information

Generation of Diffraction-Free Optical Beams Using Wrinkled Membranes

Ran Li, Hui Yi, Xiao Hu, Leng Chen, Guangsha Shi, Weimin Wang and Tian Yang

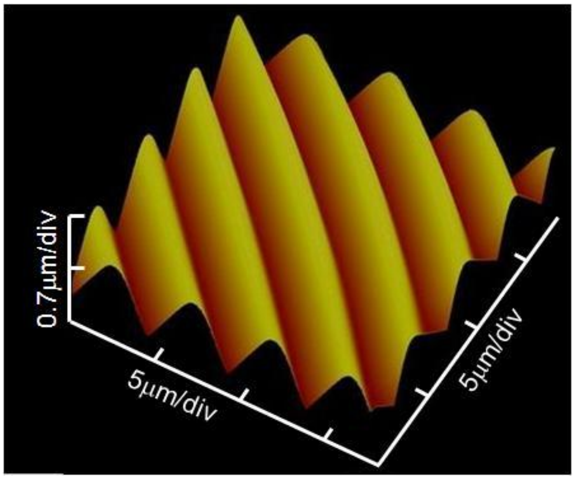


**Supplementary Figure S1. An AFM image of the wrinkles.** The sample was fabricated with the procedure as shown in Figure 1.

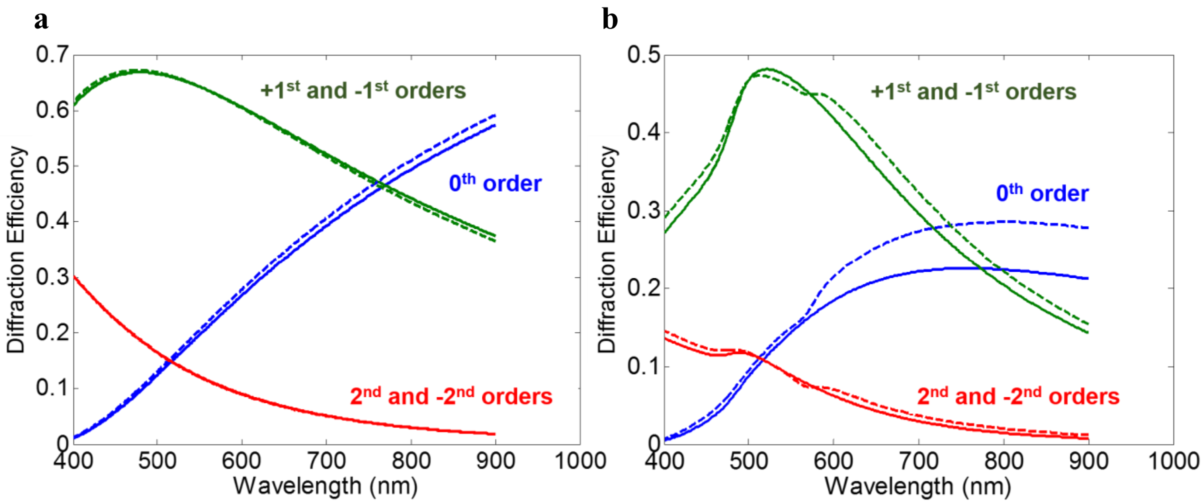


**Supplementary Figure S2**. **Diffraction efficiencies of a one dimensional sinusoidal grating.** Calculated efficiencies of the different diffraction orders of a one dimensional sinusoidal grating on a Au-PDMS bilayer membrane. The calculation is done by the Finite Difference Time Domain (FDTD) method. The PDMS layer is taken to be infinitely thick. Light is incident from the Au side and diffracts into the PDMS. The gratings have a 4.7 μm period and a 0.7 μm peak-to-peak amplitude. The refractive index of PDMS is taken to be 1.4. The refractive index of gold is taken from Supplementary Ref 42. Solid curves: TE, the incident electric field being polarized parallel to the grating grooves; dashed curves: TM, the incident electric field being polarized perpendicular to the grating grooves. (**a**) Without the Au layer. The $2^{nd}$ and $-2^{nd}$ order TM curve overlaps with the TE one and is not legible in the figure. (**b**) With a 13 nm thick Au layer.

**a** **b**

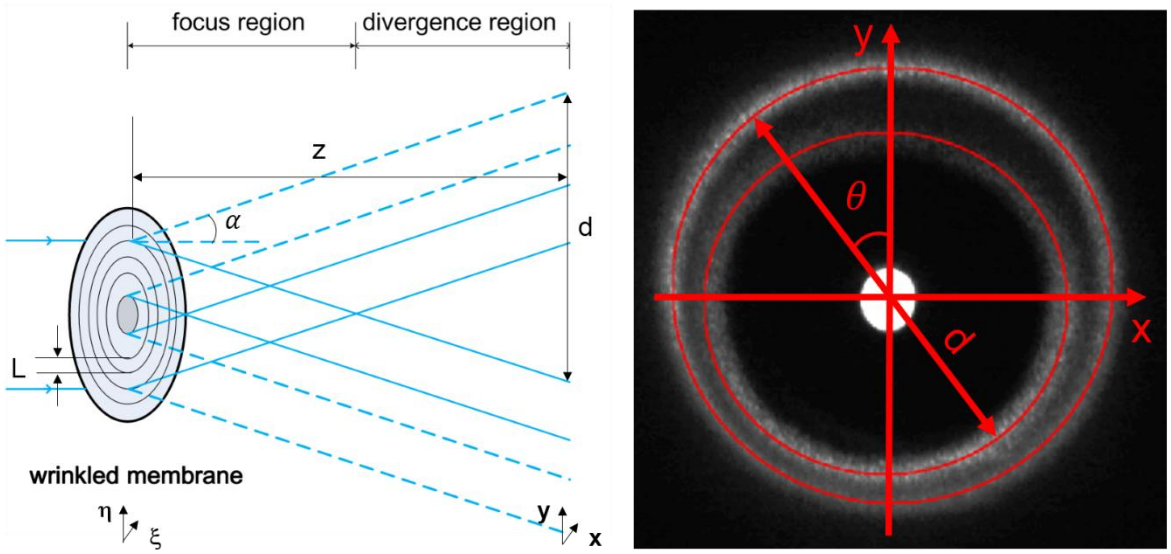


**Supplementary Figure S3. Some definitions used in focal spot profile calculation.** (**a**) Definition of some parameters in the schematic illustration of the optical experiment. (**b**) Defining the center lines of the optical rings at $z$ = 33 cm, and definition of the azimuthal angle $\theta$.

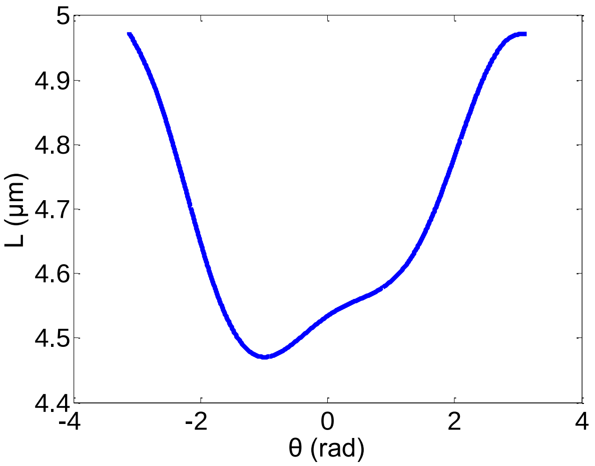


**Supplementary Figure S4. Wrinkle period at each azimuthal angle.** The plot is calculated from Supplementary Figure S3b and equation (1). $L$: wrinkle period; $\theta$: azimuthal angle.

## Supplementary References

42. Lide, D. R. *Handbook of Chemistry and Physics* (CRC Press 2008).
